# How Subtle Changes Can Make a Difference: Reproducibility in Complex Supramolecular Systems

**DOI:** 10.1002/anie.202206738

**Published:** 2022-09-05

**Authors:** Tobias Schnitzer, Marco D. Preuss, Jule van Basten, Sandra M. C. Schoenmakers, A. J. H. Spiering, Ghislaine Vantomme, E. W. Meijer

**Affiliations:** ^1^ Laboratory of Macromolecular and Organic Chemistry and Institute for Complex Molecular Systems Eindhoven University of Technology P.O. Box 513 5600 MB Eindhoven The Netherlands

**Keywords:** Complexity, Reproducibility, Solvent Effects, Supramolecular Systems

## Abstract

The desire to construct complex molecular systems is driven by the need for technological (r)evolution and our intrinsic curiosity to comprehend the origin of life. Supramolecular chemists tackle this challenge by combining covalent and noncovalent reactions leading to multicomponent systems with emerging complexity. However, this synthetic strategy often coincides with difficult preparation protocols and a narrow window of suitable conditions. Here, we report on unsuspected observations of our group that highlight the impact of subtle “irregularities” on supramolecular systems. Based on the effects of pathway complexity, minute amounts of water in organic solvents or small impurities in the supramolecular building block, we discuss potential pitfalls in the study of complex systems. This article is intended to draw attention to often overlooked details and to initiate an open discussion on the importance of reporting experimental details to increase reproducibility in supramolecular chemistry.

## Graphical abstract

The synthesis of complex systems often coincides with difficult preparation protocols and narrow windows of suitable conditions. In this Scientific Perspective, we report on unexpected observations made in our group that highlight the impact of subtle “irregularities” on supramolecular systems. We draw attention to often overlooked details and aim to initiate an open discussion on reproducibility in supramolecular chemistry.

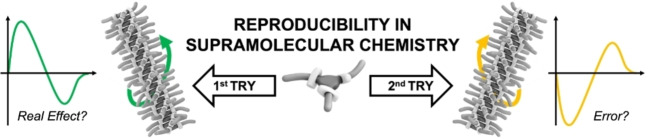

In the last decades, supramolecular chemistry has evolved into a flourishing research field at the interfaces of synthetic organic and polymer chemistry, biology, and material science. Driven by the desire to construct new materials and to better understand the fundamental characteristics of life, there is a constant effort to design and study systems based on many different components leading to increasing molecular complexity.[^1^, ^2^, ^3^, ^4^, ^5^] Complexity is described in the Cambridge dictionary as *the state of being formed from many parts* and *the state of being difficult to understand*.^[6]^ Therefore, complex systems are by definition difficult to work with and even more difficult to understand. Thus, systems that—when drawn on paper—appear simple, can exhibit complex properties because of the interplay of various components.[^5^, ^7^] This has been experienced in neighboring disciplines of supramolecular chemistry, where research over several decades has revealed subtleties that can influence the outcome of a “simple” process. The effects of subtleties are typically most pronounced in systems where a dynamic amplification process is driven by small differences in energy.[^5^, ^7^, ^8^, ^9^] An example is the highly cooperative process of crystallization in which minute energetic differences sum up to a decisive energy gain over the formation of a possibly undesired crystal lattice.[^10^, ^11^] Likewise, catalysis can lead to unexpected amplifications.[^12^, ^13^, ^14^] Only recently, reports of an unforeseen amine‐catalyzed cross‐coupling reaction—which turned out to be catalyzed by ppm traces of palladium in the amine catalyst—stimulated research on the effect of trace amounts of metals in catalytic reactions.[^15^, ^16^, ^17^, ^18^] Such examples highlight how the highly efficient catalysis of an impurity can easily outperform the intended catalyst and thus lead to unexpected results. Crystallization and catalysis are disciplines that have been explored by organic chemists for more than a century, providing in‐depth understanding of underlying effects that can bias processes. Compared to these areas, supramolecular chemistry is a much younger field. There, important effects have been described, but more frequently, undervalued subtleties are observed that can dictate the composition, the properties, and the outcome of a supramolecular system. Small effects can drastically influence supramolecular systems because of the weak interactions composing them. Identifying these subtleties and distinguishing them from potential experimental artifacts is like the search for the needle in the haystack.

In this Scientific Perspective we try to connect the dots between reproducibility issues in supramolecular chemistry and often overlooked subtleties.^[19]^ We are convinced that supramolecular chemistry has matured to a stage where well‐known assembly principles are only roughly sufficient to describe the complexity of a system. It is therefore necessary to pay attention to subtleties: tiny aspects that are easily overlooked and often not even considered when describing a system. To sensitize the reader, we highlight here experiments from our own lab that let us first struggle but which—after careful and systematic analysis—gave us an extra facet in the understanding of complex molecular systems. Derived from our lessons learned, we propose that an eye for detail combined with a rigorous and honest report of experimental details is essential for the future development of increasingly complex functional molecular systems.

## “Real” Effect or Error—A Selection of our Experiences

The cooperativity and dynamics of supramolecular systems can amplify effects based on often overlooked experimental errors. Consequently, unexpected results and reproducibility problems may occur that make us question our own competence as scientists. But when is such a surprising result an error, and when is it a real effect worth investigating? In this Scientific Perspective, we highlight several of our personal experiences with this question and describe surprising examples from our own laboratory. These examples range from simple points related to the purity of the compounds under investigation to much more delicate issues about the quality of the solvents used and how samples were prepared. Moreover, the way we study supramolecular assemblies is a delicate issue, as the structures formed are mostly highly dynamic and hence sensitive to the method of analysis. For example, solid‐state morphologies observed using atomic force microscopy can be influenced by assembly–surface rearrangements and are misleading when compared to observations made in solution.^[20]^ Further, every analytical method has its own time resolution: We encountered an illustrative example of a supramolecular system based on soft nanoparticles that was a network according to cryogenic transmission electron microscopy (cryo‐TEM), individual assemblies according to nuclear magnetic resonance (NMR) spectroscopy, and a mixture of both species according to various scattering techniques.^[21]^ In other words, describing a supramolecular structure without considering its dynamicity is too simplified. Taking these factors into account, we will distill some general remarks on how reproducibility can be increased by paying more attention to detailed descriptions of experimental aspects.

## Compound Purity

*Why is it not trivial?* Compound purity appears to be a trivial factor when it comes to reproducibility problems. However, this raises the question, when is a compound pure? In 2008, we observed a striking example in the self‐assembly of chiral oligothiophenes: Samples that would all be considered “pure” with 99.6 %, 99.9 %, and 99.9+% purity led to different assemblies based on tiny amounts of impurities.^[22]^ Here, we highlight some recent examples from our laboratory that show how hardly detectable impurities can affect supramolecular systems.

**Figure 1:**
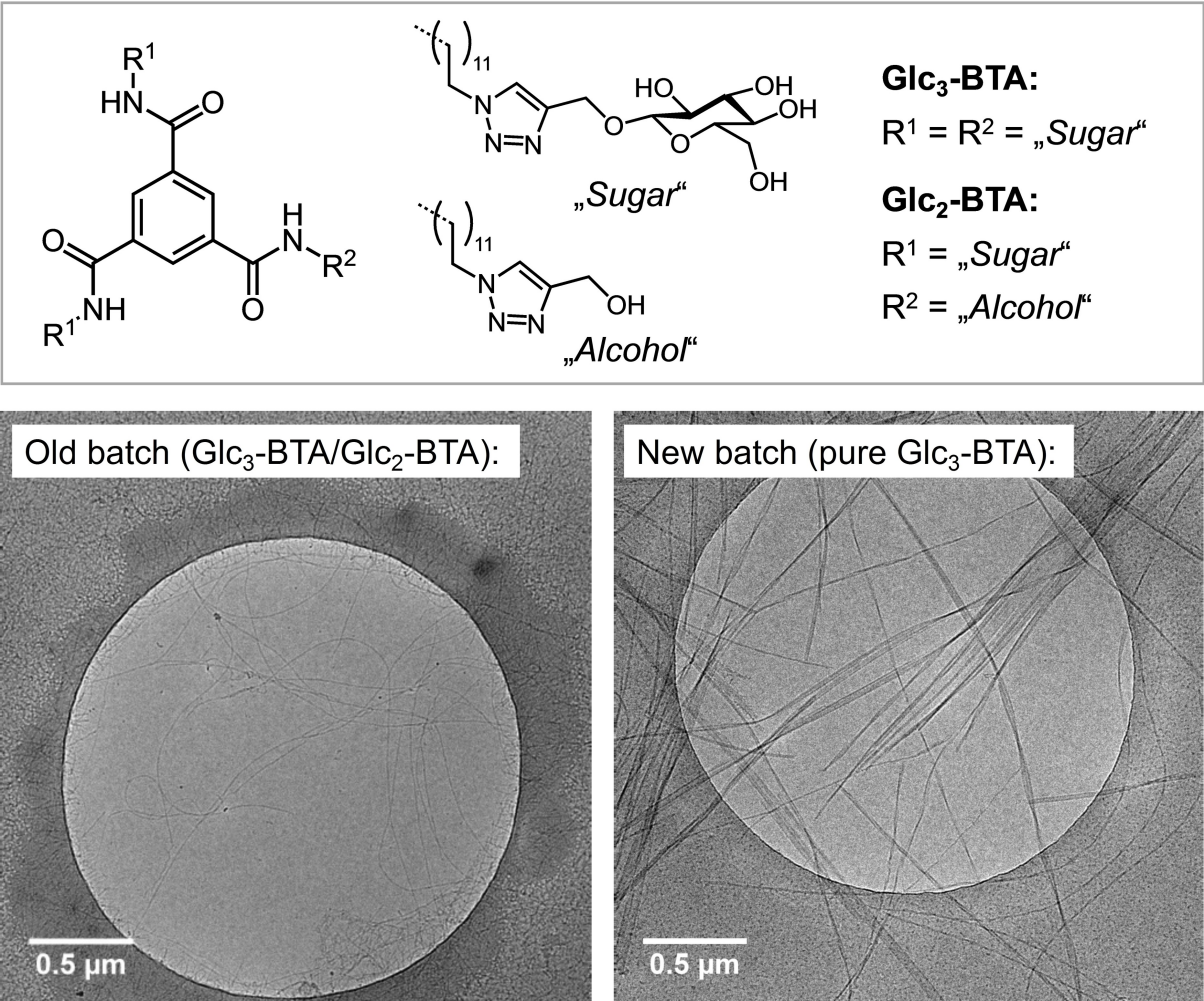
Molecular structures of Glc_3_‐BTA and Glc_2_‐BTA; cryo‐TEM of Glc_3_‐BTA/Glc_2_‐BTA mixtures (left) and pure Glc_3_‐BTA (right). Details on the analytical methods are given in the Supporting Information.

*Example 1*: Benzene‐1,3,5‐tricarboxamides (BTAs) are discotic molecules that form one‐dimensional supramolecular polymers based on threefold intermolecular hydrogen bonding.[^9^, ^23^, ^24^, ^25^] The properties of the resulting helical stacks can be tuned by the *N*‐terminal residues of the amide groups. BTAs with aliphatic side chains terminated by sugar moieties are known to polymerize supramolecularly in water, providing access to hydrogel systems.[^26^, ^27^] Recently, we studied a BTA derivative containing three terminal d‐glucose groups linked to each of the three aliphatic side chains via a triazole unit (Glc_3_‐BTA, Figure 1).^[28]^ UV/Vis spectroscopic analysis of the BTA showed supramolecular polymers at room temperature (Supporting Information Figure S3) as confirmed by thin fibers observed by cryo‐TEM (Figure 1). As the material was scarce, we decided to resynthesize Glc_3_‐BTA a few years later. While the new batch of Glc_3_‐BTA showed UV/Vis and NMR spectra (Figure S1, Figure S3) almost identical to those of the older batch, cryo‐TEM images showed—to our surprise—micrometer‐long fibers that aggregated into larger bundles (Figure 1, Figure S4). Comparison of the MS data of the two BTA batches showed minute amounts of hydrolyzed Glc_2_‐BTA in the old batch (Supporting Information Figure S2). HDX‐MS experiments revealed that “doping” Glc_3_‐BTA with a small amount of Glc_2_‐BTA has a drastic effect on the dynamics of the stacks (Supporting Information Figure S5). This unpublished example is similar to a previous study on BTAs directly linked to carbohydrates. In that case the pure, triple‐functionalized compound needed a co‐solvent to assemble into supramolecular polymers, while the water‐solubility is high enough that a co‐solvent is not required when the triple‐functionalized BTA is “doped” with small impurities of double‐functionalized BTA.^[27]^

Recently we made a somewhat related observation: while studying the properties of a comparable hydrogen‐bonded supramolecular polymer, we encountered large solubility problems in organic solvents.^[29]^ Only after the addition of a small amount of a structurally slightly different co‐monomer, did the compound dissolve. Thus, adding small amounts of “dopants or impurities” can influence the morphology, dynamics, and solubility of a supramolecular polymer.

**Scheme 1:**
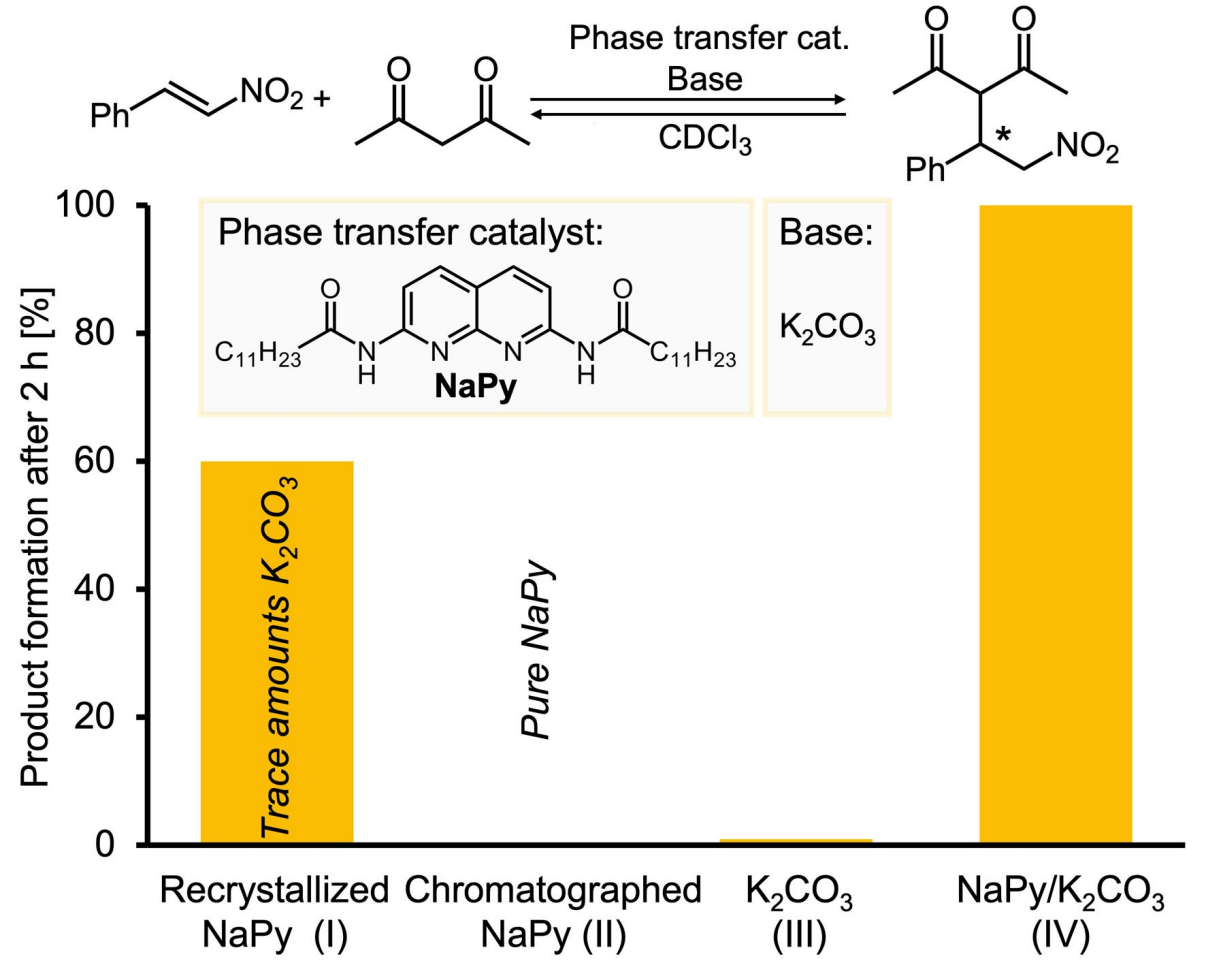
Formation of the Michael addition product between (*E*)‐nitrostyrene and 2,4‐pentadione after 2 h in CDCl_3_ catalyzed by NaPy purified by recrystallization (I, 20 mM) or column chromatography (II; 20 mM), K_2_CO_3_(III; 5 mM, hardly active) or NaPy (20 mM)/K_2_CO_3_ (5 mM) (IV). Data taken from ref. [30, 31, 32].

*Example 2*: We faced another example concerning the purity of a supramolecular building block when studying the autoregulating catalyst 2,7‐diamido‐1,8‐naphthyridine (NaPy; Scheme 1).[^30^, ^31^, ^32^] The catalytic system was based on a ureidopyrimidinone (UPy) network, which combined with the NaPy catalyst enabled a concentration‐independent activity for Michael additions. The system showed high catalytic activity for the reaction of 2,4‐pentanedione (500 mM) with (*E*)‐nitrostyrene (100 mM; in CDCl_3_, 2 h reaction time) when NaPy (20 mM) was purified by crystallization. In contrast, barely any activity was observed after a batch of NaPy was purified by column chromatography. At first glance both methods of purification resulted in seemingly pure NaPy, as indicated by identical NMR and mass spectra. Only later, we found that trace amounts of K_2_CO_3_ are key for the catalytic activity of the system: NaPy solubilizes K_2_CO_3_, which catalyzes the Michael addition resulting in a more than 100‐fold increase in reaction rate compared to the individual compounds. Traces of K_2_CO_3_ remained after recrystallization of NaPy enabling catalytic activity, while column chromatography removed the impurity causing poor reactivity.

*Lessons learned*: These selected examples showcase how reproducibility problems can easily occur due to minute amounts of impurities impacting not only the morphology but also the function of supramolecular systems. Purification of compounds typically includes the removal of side products or residual amounts of solvents. However, it is difficult to judge when a compound is pure. In most cases a compound is considered sufficiently pure when the desired mass is visible in the mass spectrum and ^1^H/^13^C NMR spectra (often accompanied by 2D correlation NMR) show the expected signals, shifts, and integrals and the absence of additional signals. As NMR spectroscopy provides no information about NMR‐inactive impurities, additional techniques (e.g. liquid chromatography‐mass spectrometry, melting point analysis, or maybe even elemental analysis) should be performed to ensure the absence of impurities. Hence, careful characterization of supramolecular building blocks by several means remains even more essential than in the past.

## Solvent Quality

In cooperative supramolecular systems, small energy differences can accumulate into large effects. An example is the amplification of asymmetry in helical supramolecular polymers.[^33^, ^34^] Only small amounts of a chiral monomer (sergeant) can bias the helical screw sense of achiral monomers (soldiers).^[33]^ In such a “sergeants‐and‐soldiers” experiment, minute differences in the optical purity of a compound can amplify into large effects. Alongside the amplification of asymmetry in copolymers, minor differences in solvation energy between a chiral solvent and achiral supramolecular monomers can drive the supramolecular aggregate to favor one helical screw sense over the other.^[35]^ Such solvent‐related amplification phenomena are not limited to chirality. Our experiences showed that also effects originating from minute amounts of an *achiral* compound can amplify. Some examples that highlight the delicacy of solvent impurities are given below.

**Figure 2:**
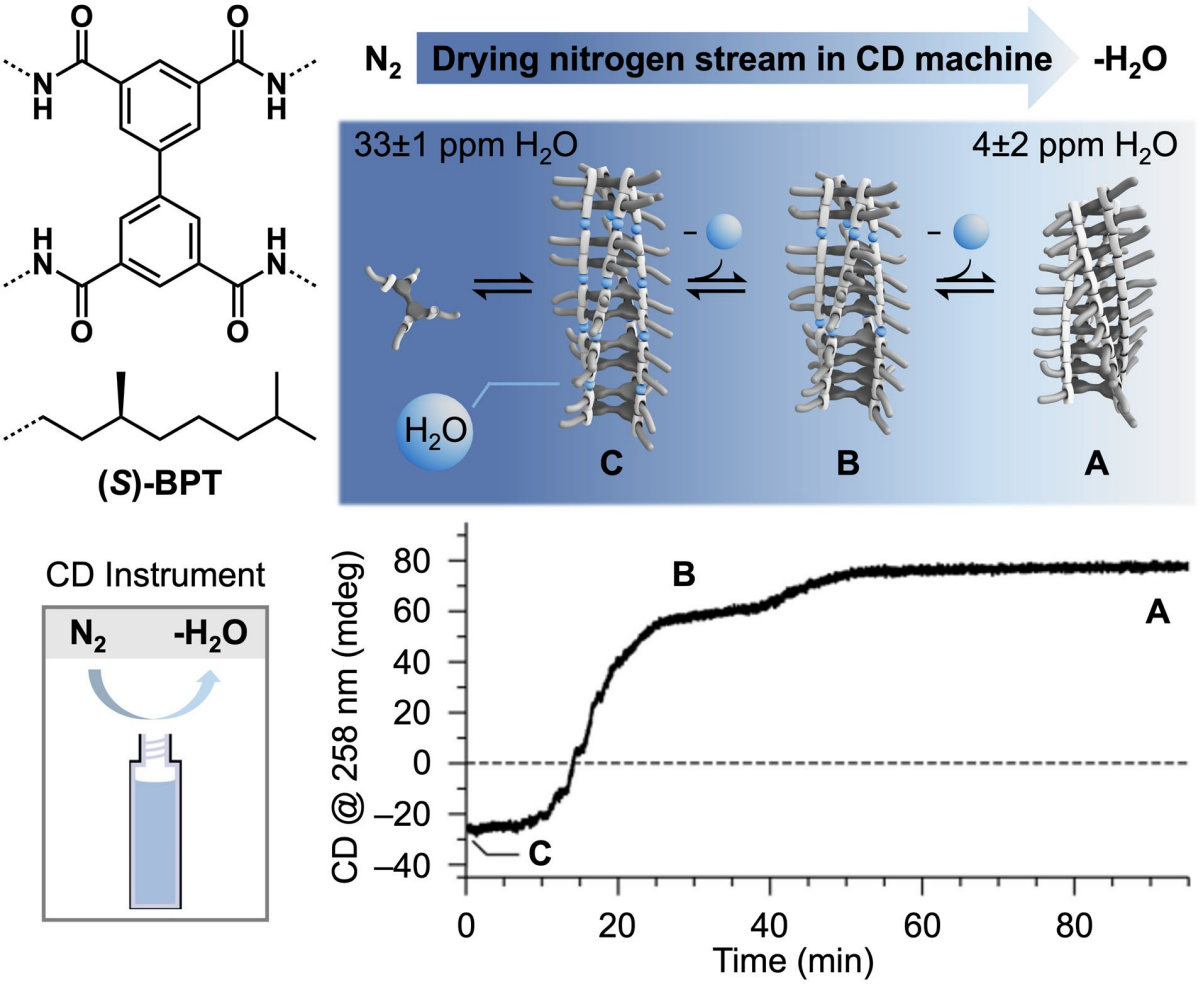
Helix inversion of (*S*)*‐*BPT and the related CD trace at 258 nm upon removal of ppm amounts of water by drying via the nitrogen stream in the CD spectrometer. Data taken from ref. [36].

*Example 1*: Recently, we came across another phenomenon that explained a series of unforeseen results. Like BTAs, also chiral bisphenyltetraamides (*S)‐*BPTs form helical supramolecular polymers (Figure 2).^[36]^ When studying the assembly of (*S)‐*BPT in methylcyclohexane (MCH) by CD spectroscopy, we observed a negative CD signal. However, the same sample showed a different, positive CD signal after remaining overnight in the CD spectrometer. This indicated the formation of a helical supramolecular polymer with opposite handedness. Also, for different batches of (*S*)*‐*BPT and MCH, these fluctuations of the CD signal kept reoccurring. Only after thorough experimental and computational analysis we found that the origin of the helix inversion is the result of small amounts of water in MCH. MCH stored under ambient conditions contains about 40 ppm of water. When samples were kept overnight in the CD spectrometer, the constant flow of dry nitrogen in the sample chamber unexpectedly reduced the amount of water in MCH, forcing inversion of the helix. Similarly, the use of different bottles of MCH (containing varying amounts of water), also led to different CD spectra originating from different supramolecular polymers with water as co‐monomer.

**Scheme 2:**
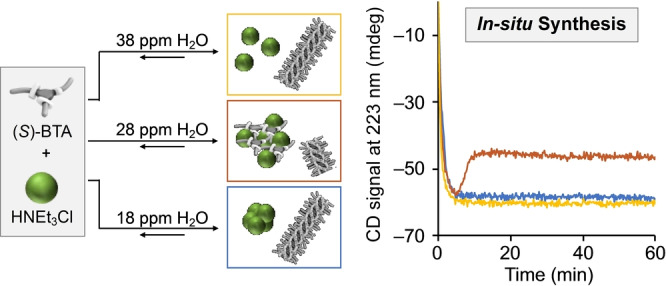
CD spectroscopic kinetic investigation of the in situ synthesis of (*S*)‐BTA‐based supramolecular polymer from trimesoic acid chloride and tetrahydrocitronellyl amine with triethylamine base in the presence of different amounts of water in MCH (solvent). Color coding of the CD spectroscopic kinetic traces: orange—38 ppm water, red—28 ppm water, orange—18 ppm water. Data taken from ref. [37].

*Example 2*: Water impurities become even more pronounced in multicomponent systems. We have demonstrated this in the in situ synthesis of (*S)‐*BTA under assembly conditions (MCH, μM concentrations) by reacting trimesoic acid chloride with tetrahydrocitronellyl amine in the presence of triethylamine base (Scheme 2).^[37]^ When we performed the reaction using MCH from different bottles, the reaction outcome changed. Following the formation of the supramolecular polymer over time by CD spectroscopy showed a strong water dependency: the expected second‐order reaction kinetics were observed in the in situ formation of the supramolecular polymer performed in “wet” (38 ppm) and “dry” (18 ppm) MCH. At intermediate water concentrations (28 ppm) unexpected kinetics were observed. We rationalized these observations with three competing assembly processes of the reaction products, (*S)‐*BTA and HNEt_3_Cl: formation of a supramolecular polymer, precipitation of HNEt_3_Cl salt, and precipitation of (*S*)*‐*BTA/HNEt_3_Cl aggregates. In “wet” MCH, (*S*)*‐*BTA and HNEt_3_Cl are well solubilized and barely interact with each other, allowing for almost quantitative polymerization. In MCH with intermediate water concentration, the interaction between (*S*)*‐*BTA and HNEt_3_Cl becomes more pronounced, leading to precipitation of the (*S*)*‐*BTA/HNEt_3_Cl aggregates. This process causes a phase separation leading to partial depolymerization and CD signal decrease. In “dry” MCH, rapid precipitation of the HNEt_3_Cl salt occurs, which yields no interference with the supramolecular polymerization. Since such phenomena are difficult to predict, it is essential to carefully report each step in our experiments and consider every potential pitfall to ensure a reproducible noncovalent synthesis of complex multicomponent systems.

**Figure 3:**
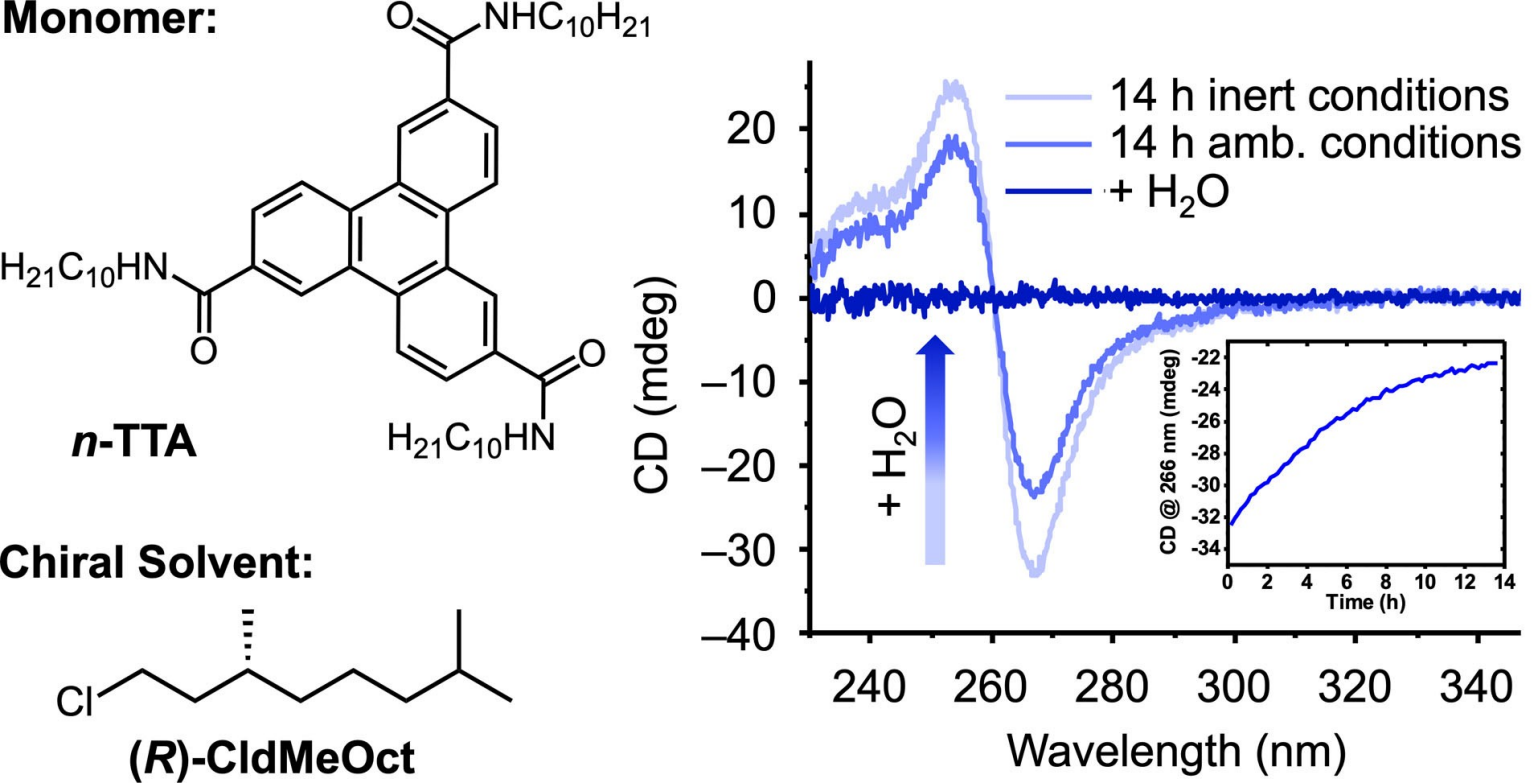
CD spectra of an aging *n*‐TTA sample in (*R*)*‐*CldMeOct after 14 h storage under inert and ambient conditions and after manual addition of water. The inset shows the decrease in CD intensity monitored at 266 nm over time (*closed* cuvette under air). Details on the analytical methods are given in the Supporting Information.

*Example 3*: We also studied the transfer of asymmetry from the chiral solvent (*R*)‐1‐chloro‐3,7‐dimethyloctane ((*R*)*‐*CldMeOct) to supramolecular polymers based on achiral 2,6,10‐triphenylenetricarboxamides (*n*‐TTA) (Figure 3).^[38]^ The chiral solvent was synthesized by chlorination of a secondary alcohol precursor using thionylchloride and pyridine followed by very careful purification via multiple distillations. Yet, CD spectra of *n*‐TTA provided inconsistent results. Samples prepared in freshly distilled solvents showed the strongest CD intensity and no significant changes were observed when the sample was kept under inert gas. In contrast, when samples from the same batch were kept under ambient conditions, a slow decrease in CD intensity over time was observed (Figure 3). We anticipated these changes to be related again to water condensed in the solvent. This hypothesis was supported by manual addition of water to the sample, which resulted in a complete loss of CD signal and a strong red‐shift in absorption indicating disassembly (Supporting Information Figure S6).

*Example 4*: Decalin is a commonly used solvent to study supramolecular polymerizations. However, the use of mixtures of *cis*‐ and *trans*‐decalin can be troublesome, as the respective diastereomeric ratio (and therewith also potential impurities) varies. In the past we experienced reproducibility issues when using different bottles of spectroscopic grade decalin coming from different or even the same vendor. We therefore compared the UV/Vis spectra of *cis*/*trans* decalin mixtures (purity ≥98 %) with that of a diasteromerically pure *cis*‐ (purity ≥98 %) and *trans*‐decalin (purity ≥98 %) (Supporting Information Figure S7). Despite the similar quality grading, strong differences in the absorption profiles are visible, which might stem from naphthalene, the precursor of decalin. As baseline correction of a sample will make such impurities invisible, their effect on the supramolecular assembly can be easily overlooked.

*Lessons learned*: Obviously, the quality of solvents is a well‐known factor in (physical) organic chemistry. Thus, everyone uses spectroscopy‐grade solvents. However, our examples show that even such high‐grade solvents are not always good enough to achieve high‐quality reproducibility. As supramolecular systems are typically studied in μM concentrations, already minute amounts of solvent impurities (e.g. water or byproducts of the solvent synthesis) can be stoichiometric with respect to the components of the supramolecular system. Thus, a careful analysis (and if necessary purification) of the solvents is as important as the analysis of the compounds that are investigated. Moreover, for specific systems it is even recommended to perform the experiments in a glove box or otherwise humidity‐controlled conditions.

## Sample Preparation

**Scheme 3:**
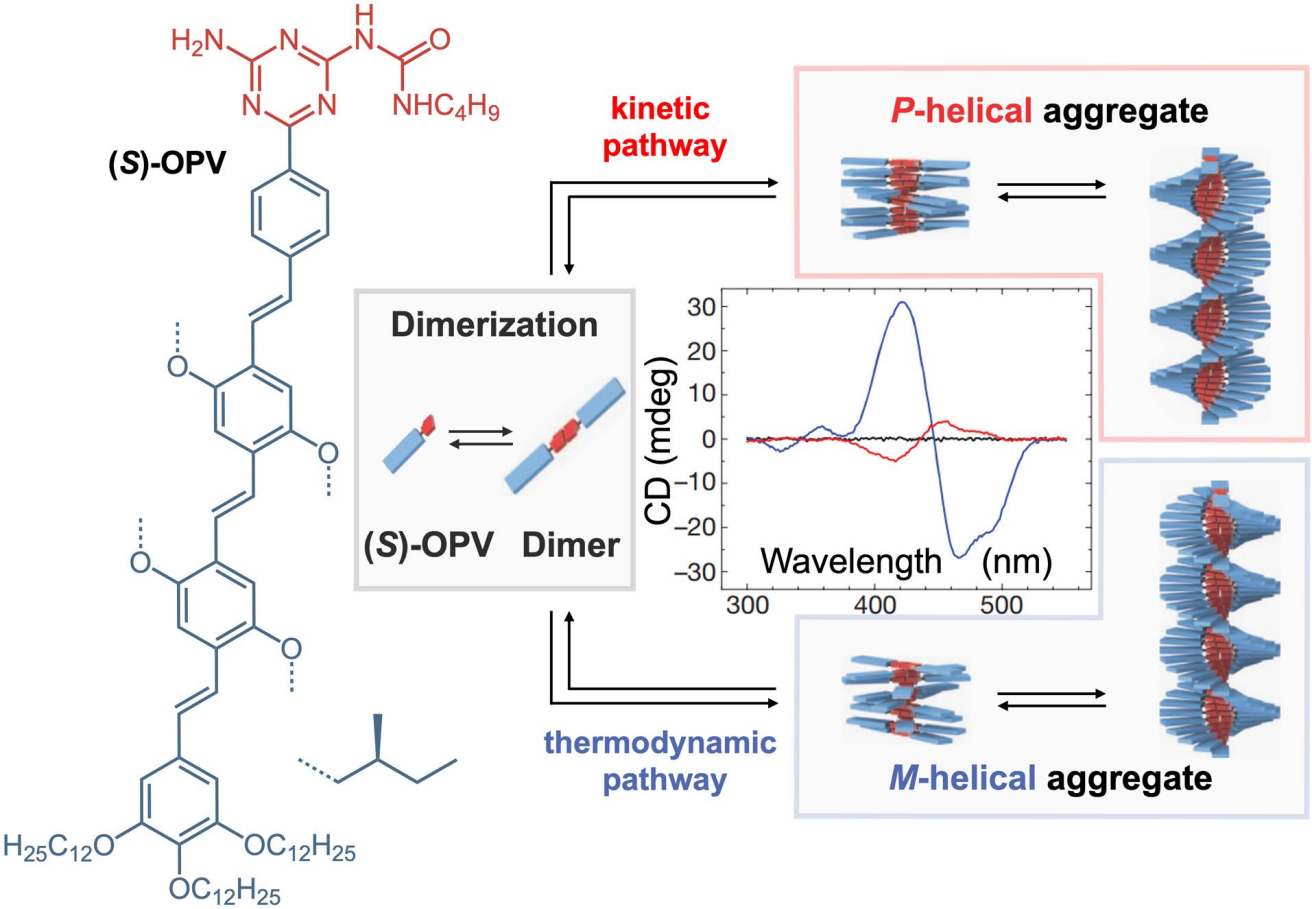
Pathway complexity of the (*S*)*‐*OPV dimer, which undergoes kinetically or thermodynamically favored formation of the *P*‐ or *M*‐helical supramolecular polymer. The CD spectra of (*S*)*‐*OPV show the *M*‐ (blue line) and *P*‐helical aggregates (red line), and the molecular dissolved state (black line). Data taken from ref. [39].

Besides the sample quality, also sample preparation can have drastic effects on the resulting supramolecular structures. A remarkable example is the polymerization of the chiral oligo(*para*‐phenylenevinylene) (*S*)*‐*OPV (Scheme 3).^[39]^ This structure forms hydrogen‐bonded dimers that assemble into helical stacks in alkanes. The assembly properties including the helicity of the polymer were studied by CD spectroscopy. Surprisingly when the polymers were prepared, different CD spectra were repeatedly obtained indicating the presence of different species. Only when a very careful preparation protocol was followed could the spectroscopic data be reproduced. In‐depth spectroscopic and computational analysis revealed the origin of the strange assembly behavior: (*S*)*‐*OPV can polymerize into two pseudo‐enantiomeric helical structures. Under thermodynamic control, the (*M*)‐helical polymer is formed, under kinetic control the (*P*)‐helical polymer. This phenomenon—referred to as *pathway complexity* ^[39]^—is reminiscent of observations in the Diels–Alder reaction, which can also form two different reaction products depending on the applied conditions.^[40]^ Typically, the *endo*‐configured product is obtained at room temperature (kinetic product) and the *exo*‐configured product is formed when the reaction is performed under reflux (thermodynamic product). However, as the supramolecular polymerization is dynamic and relies on weak interactions, it is much more sensitive to the reaction conditions compared to the covalent product formation in the Diels–Alder reaction. As a result, variations of only a few °C of temperature or tiny changes in the heating/cooling rate have a major impact on the resulting supramolecular structures due to unexpected pathway complexity. Like pathway complexity, also stereomutation and supercoiling are prone to misinterpretation.[^41^, ^42^, ^43^] Even small concentration differences can lead to different CD spectra e.g. based on concentration‐dependent formation of chiral superstructures with opposite handedness.

Similar to supramolecular polymers, kinetic traps are also reported in the area of π‐conjugated covalent polymers.[^44^, ^45^] In many cases these polymers aggregate in an uncontrolled fashion during the final process after purification, e.g. solvent removal by rotary evaporation. These stable aggregates cannot be disassembled when the polymer is re‐processed and not even when a good solvent is used. Consequently, ill‐defined morphologies are prepared. Only (solvent) annealing at higher temperatures or the use of co‐solvents in the sample preparation can solve this issue to access polymers with the desired morphology.^[46]^

*Lessons learned*: Human inconsistencies are hardly avoidable. Person A might prepare a solution of a given compound by heating the mixture, person B rather stirs overnight, and person C prefers sonication for a few hours. One is inclined to assume that the result—a homogeneous solution—is the same. Yet, different equilibration techniques can trap systems in different kinetic and thermodynamic states resulting in different properties. Thus, to minimize reproducibility issues, it is essential to rigorously report, even for seemingly unimportant steps, all the details of the experimental assembly protocols.

Furthermore, even trivial errors can become important: Were clean cuvettes without scratches and with compatible rubber seals used? Was the temperature control of the measured sample accurate?^[47]^ Hence, was there sufficient heat transfer between sample and temperature unit and was the sample properly stirred? Although these points seem superficial, they often can explain unexpected observations on supramolecular systems.

## Data Misinterpretation

Finally, we would like to dedicate one short paragraph to CD spectroscopy, which is commonly used to characterize supramolecular systems. Fascinating examples are reported, which show significant Cotton effects of achiral compounds in CD spectra due to chiral mechanical forces.[^48^, ^49^] Furthermore in a stochastic process, an achiral compound can form sometimes one, sometimes the other enantiomeric supramolecular polymer. Only if the experiment is repeated multiple times will a 50 : 50 ratio of both enantiomeric forms be observed.^[50]^ However, such processes are rare. The reason for CD signals of achiral compounds is often anisotropy of the sample.[^51^, ^52^] Such samples are prone to misinterpretation, as linear dichroism or linear birefringence can cause apparent CD signals.[^53^, ^54^] These effects are often linked to alignment of the fibers by temperature gradients and convective flow or incomplete dissolution of the sample. Thus, it is essential to perform experiments only with fully dissolved, homogeneous samples to ensure reproducible measurements. Moreover, similar precautions have to be taken when measuring CD spectra of thin films.^[55]^

## Conclusions

Supramolecular chemistry has reached a level of sophistication and complexity at which not only major effects but also subtleties can play a drastic role in determining the properties of systems. Thus, the differentiation of a “real effect” from a systematic error becomes essential. While collecting an exhaustive list of potential pitfalls in supramolecular chemistry is almost impossible, we have aimed here to sensitize scientists to easily underappreciated factors. Although exemplified with hydrogen‐bonded supramolecular polymers, the purity of a building block, the purity of a spectroscopic‐grade solvent, or the importance of exact adjustment of reaction conditions can challenge us with reproducibility issues in all areas of supramolecular chemistry. It is therefore essential to rethink the way we currently perform supramolecular chemistry and construct complex systems. Maybe we need to perform our reactions in a glove box to prevent interference of water or air. Should we repeat our experiments with reagents from different vendors to exclude—or at least reduce—the chance for biases from impurities? Should we distill every solvent and repurify every substance prior to use? Maybe it is necessary to automate the synthesis of supramolecular systems to prevent experimental variations and eliminate human errors as much as possible.^[56]^ Moreover, statistical analysis of repeated reactions can give insights on errors and biased measurements versus “real effects”, although for many points discussed here, a statistical analysis is very hard to perform. Another idea is to transfer the concept of the journal *Organic Syntheses* to a journal *Noncovalent Syntheses*, for publishing reliable protocols where each set of directions has been repeated, and every experiment has been carried out in at least two laboratories. Regardless of the way the field of supramolecular chemistry will evolve, a first step to prevent reproducibility issues should be the honest report of experiments. Studies that only work with a certain bottle of solvent, experiments that failed ten times before suddenly starting to produce satisfying results, and even negative results should be carefully reported. In this way, our community can learn from each other and hopefully collectively tackle the challenges that come along with the synthesis of complex molecular systems.

## Author Contributions

T.S., M.D.P., and E.W.M. conceived and designed the overall project. J.v.B. performed the decalin solvent study. S.M.C.S. and A.J.H.S. performed the experiments on the Glc‐BTAs. T.S., M.D.P., G.V., and E.W.M. prepared the manuscript with contributions from all authors.

## Conflict of interest

The authors declare no conflict of interest.

## Supporting information

As a service to our authors and readers, this journal provides supporting information supplied by the authors. Such materials are peer reviewed and may be re‐organized for online delivery, but are not copy‐edited or typeset. Technical support issues arising from supporting information (other than missing files) should be addressed to the authors.

Supporting Information

## Data Availability

The data that support the findings of this study are found in the Supporting Information of this article.

## References

[1] J.-F. Lutz , J.-M. Lehn , E. W. Meijer , K. Matyjaszewski , Nat. Rev. Mater. 2016, 1, 16024.

[2] B. L. Feringa , Angew. Chem. Int. Ed. 2017, 56, 11060; 10.1002/anie.201702979 28851050

[3] J. F. Stoddart , Angew. Chem. Int. Ed. 2017, 56, 11094; 10.1002/anie.201703216 28815900

[4] J. P. Sauvage , Angew. Chem. Int. Ed. 2017, 56, 11080; 10.1002/anie.201702992 28632333

[5] G. Vantomme , E. W. Meijer , Science 2019, 363, 1396. 30923212 10.1126/science.aav4677

[6] Cambridge Dictionary, Cambridge University Press, definition of “complexity,” **2021**.

[7] T. Schnitzer , G. Vantomme , ACS Cent. Sci. 2020, 6, 2060. 33274282 10.1021/acscentsci.0c00974 PMC7706085

[8] T. Aida , E. W. Meijer , S. I. Stupp , Science 2012, 335, 813. 22344437 10.1126/science.1205962 PMC3291483

[9] C. Kulkarni , E. W. Meijer , A. R. A. A. Palmans , Acc. Chem. Res. 2017, 50, 1928. 28692276 10.1021/acs.accounts.7b00176 PMC5559720

[10] G. R. Desiraju , The Crystal as a Supramolecular Entity, Wiley, Weinheim, Germany, 1996.

[11] M. Olbrycht , M. Balawejder , I. Poplewska , H. Lorenz , A. Seidel-Morgenstern , W. Piatkowski , D. Antos , Cryst. Growth Des. 2019, 19, 1786.

[12] N. E. Leadbeater , M. Marco , J. Org. Chem. 2003, 68, 5660. 12839459 10.1021/jo034230i

[13] R. K. Arvela, N. E. Leadbeater, S. M. S., V. A. Williams, P. Granados, R. D. Singer, *J. Org. Chem*. **2005**, *70*, 161. 10.1021/jo048531j 15624918

[14] I. Thomé , A. Nijs , C. Bolm , Chem. Soc. Rev. 2012, 41, 979. 22218700 10.1039/c2cs15249e

[15] L. Xu , F. Y. Liu , Q. Zhang , W. J. Chang , Z. L. Liu , Y. Lv , H. Z. Yu , J. Xu , J. J. Dai , H. J. Xu , Nat. Catal. 2021, 4, 71.

[16] J. K. Vinod , A. K. Wanner , E. I. James , K. Koide , Nat. Catal. 2021, 4, 999.

[17] Z. Novák , R. Adamik , J. T. Csenki , F. Béke , R. Gavaldik , B. Varga , B. Nagy , Z. May , J. Daru , Z. Gonda , G. L. Tolnai , Nat. Catal. 2021, 4, 991.

[18] M. Avanthay , R. B. Bedford , C. S. Begg , D. Böse , J. Clayden , S. A. Davis , J. C. Eloi , G. P. Goryunov , I. V. Hartung , J. Heeley , K. A. Khaikin , M. O. Kitching , J. Krieger , P. S. Kulyabin , A. J. J. Lennox , R. Nolla-Saltiel , N. E. Pridmore , B. J. S. Rowsell , H. A. Sparkes , D. V. Uborsky , A. Z. Voskoboynikov , M. P. Walsh , H. J. Wilkinson , Nat. Catal. 2021, 4, 994.

[19] R. G. Bergman , R. L. Danheiser , Angew. Chem. Int. Ed. 2016, 55, 12548; 10.1002/anie.201606591 27558212

[20] P. Jonkheijm , F. J. M. Hoeben , R. Kleppinger , J. Van Herrikhuyzen , A. P. H. J. Schenning , E. W. Meijer , J. Am. Chem. Soc. 2003, 125, 15941. 14677986 10.1021/ja0383118

[21] T. M. Hermans , M. A. C. Broeren , N. Gomopoulos , P. Van Der Schoot , M. H. P. Van Genderen , N. A. J. M. Sommerdijk , G. Fytas , E. W. Meijer , Nat. Nanotechnol. 2009, 4, 721. 19893514 10.1038/nnano.2009.232

[22] M. Wolffs , P. A. Korevaar , P. Jonkheijm , O. Henze , W. J. Feast , A. P. H. J. Schenning , E. W. Meijer , Chem. Commun. 2008, 4613. 10.1039/b809560d 18815701

[23] L. Brunsveld , A. P. H. J. Schenning , M. A. C. Broeren , H. M. Janssen , J. A. J. M. Vekemans , E. W. Meijer , Chem. Lett. 2000, 29, 292.

[24] S. Cantekin , T. F. A. de Greef , A. R. A. Palmans , Chem. Soc. Rev. 2012, 41, 6125. 22773107 10.1039/c2cs35156k

[25] P. J. M. Stals , J. F. Haveman , R. Martín-Rapún , C. F. C. Fitié , A. R. A. Palmans , E. W. Meijer , J. Mater. Chem. 2009, 19, 124.

[26] C. M. A. Leenders , G. Jansen , M. M. M. Frissen , R. P. M. Lafleur , I. K. Voets , A. R. A. Palmans , E. W. Meijer , Chem. Eur. J. 2016, 22, 4608. 26890574 10.1002/chem.201504762

[27] S. I. S. Hendrikse , L. Su , T. P. Hogervorst , R. P. M. Lafleur , X. Lou , G. A. Van Der Marel , J. D. C. Codee , E. W. Meijer , J. Am. Chem. Soc. 2019, 141, 13877. 31387351 10.1021/jacs.9b06607 PMC6733156

[28] S. M. C. Schoenmakers , C. M. A. Leenders , R. P. M. Lafleur , X. Lou , E. W. Meijer , G. M. Pavan , A. R. A. Palmans , Chem. Commun. 2018, 54, 11128. 10.1039/c8cc04818e 30225478

[29] N. J. Van Zee , M. F. J. Mabesoone , B. Adelizzi , A. R. A. Palmans , E. W. Meijer , J. Am. Chem. Soc. 2020, 142, 20191. 33169999 10.1021/jacs.0c10456 PMC7705959

[30] F. Rodríguez-Llansola , E. W. Meijer , J. Am. Chem. Soc. 2013, 135, 6549. 23548154 10.1021/ja4006833

[31] F. Rodríguez-Llansola , E. W. Meijer , J. Am. Chem. Soc. 2015, 137, 8654. 26121009 10.1021/jacs.5b04626

[32] A. J. P. Teunissen , R. J. C. van der Haas , J. A. J. M. Vekemans , A. R. A. Palmans , E. W. Meijer , Bull. Chem. Soc. Jpn. 2016, 89, 308.

[33] A. R. A. Palmans , J. A. J. M. Vekemans , E. E. Havinga , E. W. Meijer , Angew. Chem. Int. Ed. Engl. 1997, 36, 2648;

[34] M. M. J. Smulders , A. P. H. J. Schenning , E. W. Meijer , J. Am. Chem. Soc. 2007, 130, 606. 10.1021/ja075987k 18081281

[35] M. L. Ślęczkowski , M. F. J. Mabesoone , P. Ślęczkowski , A. R. A. Palmans , E. W. Meijer , Nat. Chem. 2021, 13, 200. 33257888 10.1038/s41557-020-00583-0

[36] N. J. Van Zee , B. Adelizzi , M. F. J. Mabesoone , X. Meng , A. Aloi , H. Zha , M. Lutz , I. A. W. Filot , A. R. A. Palmans , E. W. Meijer , Nature 2018, 558, 100. 29849144 10.1038/s41586-018-0169-0

[37] T. Schnitzer , M. F. J. Mabesoone , S. A. H. Jansen , G. Vantomme , E. W. Meijer , Angew. Chem. Int. Ed. 2022, 61, e202206729; 10.1002/anie.202206729 PMC9544088 35763321

[38] A. K. Mondal , M. D. Preuss , M. L. Śléczkowski , T. K. Das , G. Vantomme , E. W. Meijer , R. Naaman , J. Am. Chem. Soc. 2021, 143, 7189. 33926182 10.1021/jacs.1c02983 PMC8297732

[39] P. A. Korevaar , S. J. George , A. J. Markvoort , M. M. J. Smulders , P. A. J. Hilbers , A. P. H. J. Schenning , T. F. A. De Greef , E. W. Meijer , Nature 2012, 481, 492. 22258506 10.1038/nature10720

[40] L. Kürti , B. Czakó , Strategic Applications of Named Reactions in Organic Synthesis: Background and Detailed Mechanisms, Elsevier Academic Press, Amsterdam, 2005.

[41] A. Lohr , M. Lysetska , F. Würthner , Angew. Chem. Int. Ed. 2005, 44, 5071; 10.1002/anie.200500640 16013073

[42] M. Hifsudheen , R. K. Mishra , B. Vedhanarayanan , V. K. Praveen , A. Ajayaghosh , Angew. Chem. Int. Ed. 2017, 56, 12634; 10.1002/anie.201707392 28799691

[43] M. F. J. Mabesoone , A. J. Markvoort , M. Banno , T. Yamaguchi , F. Helmich , Y. Naito , E. Yashima , A. R. A. Palmans , E. W. Meijer , J. Am. Chem. Soc. 2018, 140, 7810. 29886728 10.1021/jacs.8b02388 PMC6026832

[44] P. Leclère , M. Surin , P. Viville , R. Lazzaroni , A. F. M. Kilbinger , O. Henze , W. J. Feast , M. Cavallini , F. Biscarini , A. P. H. J. Schenning , E. W. Meijer , Chem. Mater. 2004, 16, 4452.

[45] M. M. Bouman , E. W. Meijer , Adv. Mater. 1995, 7, 385.

[46] C. Kulkarni , R. H. N. Curvers , G. Vantomme , D. J. Broer , A. R. A. Palmans , S. C. J. Meskers , E. W. Meijer , Adv. Mater. 2021, 33, 2005720. 10.1002/adma.202005720 PMC11468155 33270297

[47] Note: Sample cooling based on tap water can struggle to follow programmed temperature gradients as the water temperature varies with the time of the year (summer vs. winter). Thus, external thermostats for the cooling water ensure constant water temperatures.

[48] J. M. Ribó , J. Crusats , F. Sagués , J. Claret , R. Rubires , Science 2001, 292, 2063. 11408653 10.1126/science.1060835

[49] A. Sorrenti , Z. El-Hachemi , O. Arteaga , A. Canillas , J. Crusats , J. M. Ribo , Chem. Eur. J. 2012, 18, 8820. 22678975 10.1002/chem.201200881

[50] A. Adawy , Symmetry 2022, 14, 292.

[51] M. Wolffs , S. J. George , Ž. Tomović , S. C. J. Meskers , A. P. H. J. Schenning , E. W. Meijer , Angew. Chem. Int. Ed. 2007, 46, 8203; 10.1002/anie.200703075 17886314

[52] A. Tsuda , M. A. Alam , T. Harada , T. Yamaguchi , N. Ishii , T. Aida , Angew. Chem. Int. Ed. 2007, 46, 8198; 10.1002/anie.200703083 17768756

[53] R. L. Disch , D. I. Sverdlik , Anal. Chem. 1969, 41, 82.

[54] Y. Shindo , Y. Ohmi , J. Am. Chem. Soc. 1985, 107, 91.

[55] G. Albano , G. Pescitelli , L. Di Bari , Chem. Rev. 2020, 120, 10145. 32892619 10.1021/acs.chemrev.0c00195

[56] L. Porwol , D. J. Kowalski , A. Henson , D. L. Long , N. L. Bell , L. Cronin , Angew. Chem. Int. Ed. 2020, 59, 11256; 10.1002/anie.202000329 PMC7384156 32419277

