# Supplementary material for: How Subtle Changes Can Make a Difference: Reproducibility in Complex Supramolecular Systems
Source: Angew Chem Int Ed Engl. 2022 Sep 5;61(41):e202206738. doi: 10.1002/anie.202206738 (PMC9825988; doi:10.1002/anie.202206738)
Supplement: Supplementary file 1 — Supporting Information [file ANIE-61-0-s001.pdf]

Angewandte Chemie
Eine Zeitschrift der Gesellschaft Deutscher Chemiker

Supporting Information

How Subtle Changes Can Make a Difference: Reproducibility in Complex Supramolecular Systems

*T. Schnitzer, M. D. Preuss, J. van Basten, S. M. C. Schoenmakers, A. J. H. Spiering, G. Vantomme, E. W. Meijer**

Supporting Information

Contents

1 Materials and Methods S2

1.1 General Considerations S2

1.2 Characterization Methods S2

2 Experimental Data S4

2.1 Characterization of **Glc_3-BTA**/**Glc_2-BTA** and Pure **Glc_3-BTA** S4

2.1.1 ^{1}H-NMR and LC-MS Analysis S4

2.1.2 Sample preparation of **Glc_3-BTA** in water S5

2.1.3 UV-vis Spectroscopic Analysis S6

2.1.4 Cryo-TEM Measurements S7

2.1.5 HDX-MS Measurements S7

2.2 Impact of Water on the Self-Assembly of ***n*-TTA** in **(*R*)-CldMeOct** S9

2.2.1 Sample Preparation S9

2.2.2 UV-vis Spectroscopic Analysis S9

2.3 UV-vis Spectroscopic Analysis of Different Decalins S10

3 References S11

1 Materials and Methods

1.1 General Considerations

This supporting information contains previously unpublished data.

All used materials were purchased from commercial sources and were used without further purification unless otherwise noted. Solvents for spectroscopic measurements were purchased in spectroscopic grade quality.

1.2 Characterization Methods

NMR analysis was carried out using Bruker Mercury Vx 400 MHz spectrometers using deuterated solvents purchased from Cambridge Isotope Laboratories. The NMR spectra were processed using MestReNova x64 14.01. (Mestrelab Research S.L.).

Liquid chromatography mass spectroscopy (LC-MS) spectra were acquired using a device consisting of multiple components: Shimadzu SCL-10 A VP system controller with Shimadzu LC-10AD VP liquid chromatography pumps with an Alltima C18 3 u (50 x 2.1 mm) reversed-phase column and gradients of water, a Shimadzu DGU 20A3 prominence degasser, a Thermo Finnigan surveyor auto sampler, a Thermo Finnigan surveyor PDA detector and a Thermo Scientific LCW Fleet. All samples were dissolved in 1:1 H_2O:ACN in ca. 0.1 mg/mL concentration.

Ultraviolet (UV) absorbance spectra were recorded on a JASCO V-650 UV-Vis spectrometer equipped with a JASCO ETCT-762 temperature controller or on a JASCO V-750 UV-Vis spectrometer equipped with a PAC-743 multi-cuvette holder and JASCO ETCT-762 temperature controller with a temperature range of 263 K to 383 K. The temperature controllers were connected to external circulating thermostat JASCO CTU-100 as cooling water source. Measurements were performed using HELLMA Quartz cuvettes with an optical pathlength of 10 mm. All measurements were baseline corrected.

Electronic circular dichroism (ECD) measurements were performed using JASCO J-815 CD spectrometer equipped with a JASCO Peltier PFD-425S/15 with a temperature range of 263 K to 383 K using the following settings; sensitivity: Standard, D.I.T: 0.25 s, bandwidth: 1 nm, scanning speed: 50 nm/min, data pitch: 0.2 nm. All ECD measurements were performed using HELLMA quartz cuvettes with an optical pathlength of 1 mm and 9 mm metal spacer as heat bridge. The temperature controllers were connected to external circulating thermostat JASCO CTU-100 as cooling water source. All measurements were baseline corrected.

Cryogenic transmission electron microscopy (cryoTEM) images were made of vitrified samples with a concentration of 500 µM. Vitrified films were prepared in a 'Vitrobot'

instrument (FEI Vitrobot^{TM} Mark IV, FEI Company) at 22 °C and at a relative humidity of 100%. In the preparation chamber of the 'Vitrobot', 3 µL samples were applied on Quantifoil grids (R 2/2, Quantifoil Micro Tools GmbH), which were surface plasma treated just prior to use (Cressington 208 carbon coater operating at 5 mA for 40 s). Excess sample was removed by blotting using filter paper for 3 s with a blotting force of -1, and the thin film thus formed was plunged (acceleration about 3 g) into liquid ethane just above its freezing point. Vitrified films were transferred into the vacuum of a CryoTITAN equipped with a field emission gun that was operated at 300 kV, a post-column Gatan energy filter, and a 2048 x 2048 Gatan CCD camera. Virtrified films were observed in the CryoTITAN microscope at temperatures below -170 °C. Micrographs were taken at low dose conditions, starting at a magnification of 6500 with a defocus setting of -40 µm or at a magnification of 24000 with a defocus setting of -10 µm.

Hydrogen deuterium exchange experiments with electrospray ionization mass spectrometry were carried out using a Xevo^{TM} G2 QTof mass spectrometer (Waters) with a capillary voltage of 2.7 kV, a cone voltage of 80 V and an extraction cone voltage of 4.0 V. The source temperature was set at 100 °C, the desolvation temperature at 400 °C, and the cone gas flow at 10 L/h and the desolvation gas flow at 500 L/h. The sample solutions subjected to H/D exchange were introduced into the mass spectrometer using a Harvard syringe pump (11 Plus, Harvard Apparatus) at a flow rate of 50 µL/min. The signal was left to equilibrate for 1 minute before starting the measurement and each measurement was averaged over 1 minute to account for instabilities in the signal. Spectra were recorded in centroid mode and the intensity of the peaks is used for the calculations as described below. Before each measurement, the system was calibrated with a 0.05% H_3PO_4 solution in 1:1 H_2O:ACN. Isotope patterns for calculation were determined with IsoPro software.

Solvent addition was performed using Gilson MICROMAN Positive-Displacement Pipets (range: 3 µL - 25 µL, 25 µL – 100 µL, 50 µL – 250 µL and 100 µL – 1000 µL). Compounds were weighted using a Sartorius Lab Instrument microbalance (d=0,0001 mg).

2 Experimental Data

2.1 Characterization of **Glc3-BTA**/**Glc2-BTA** and Pure **Glc3-BTA**

Glc3-BTA was synthesized as previously published.[1]

2.1.1 ^{1}H-NMR and LC-MS Analysis

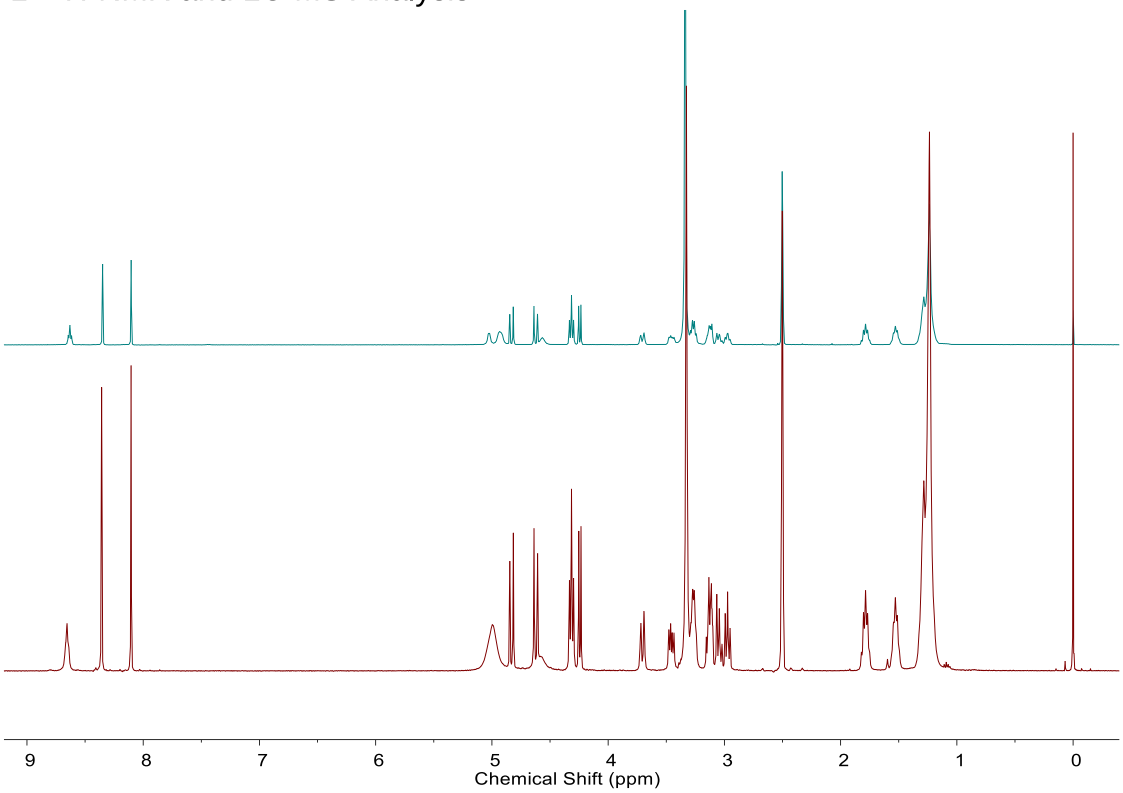

Figure S1: ^{1}H-NMR of **Glc3-BTA/Glc2-BTA** (bottom) and **Glc3-BTA** (top) in DMSO-d_6 (10 mg/mL).

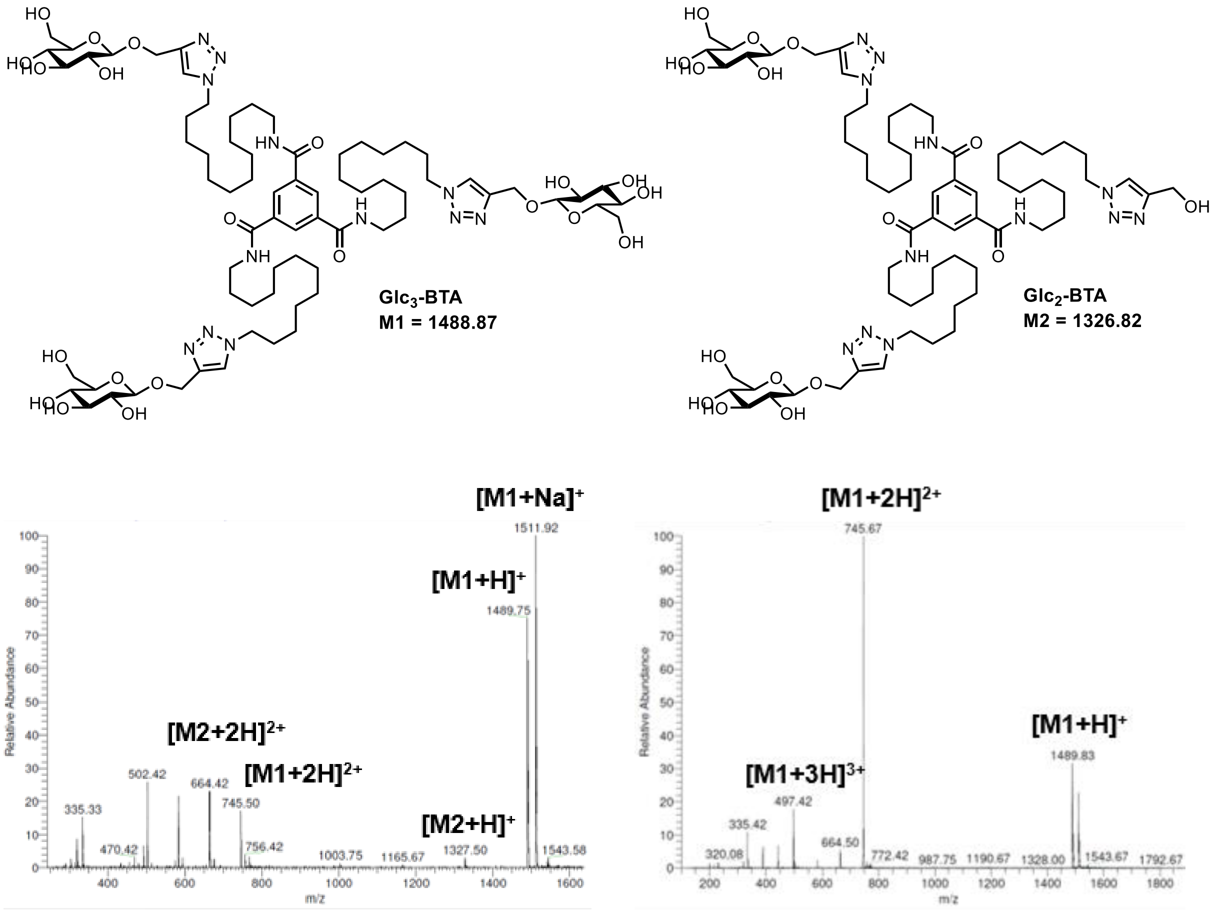

Figure S2: Mass spectrum of the old (left) and new (right) batch of the BTA. The old batch shows signals indicative of **Glc$_2$-BTA**.

2.1.2 Sample preparation of **Glc$_3$-BTA** in water

The solid material was weighed into a glass vial equipped with a magnetic stirring bar. MQ-water was added to obtain the desired concentration. The sample was subsequently stirred at 80 °C for 15 minutes and the hot and hazy sample was vortexed immediately afterwards for 15 seconds. All samples were left to equilibrate at room temperature overnight before any measurement.

2.1.3 UV-vis Spectroscopic Analysis

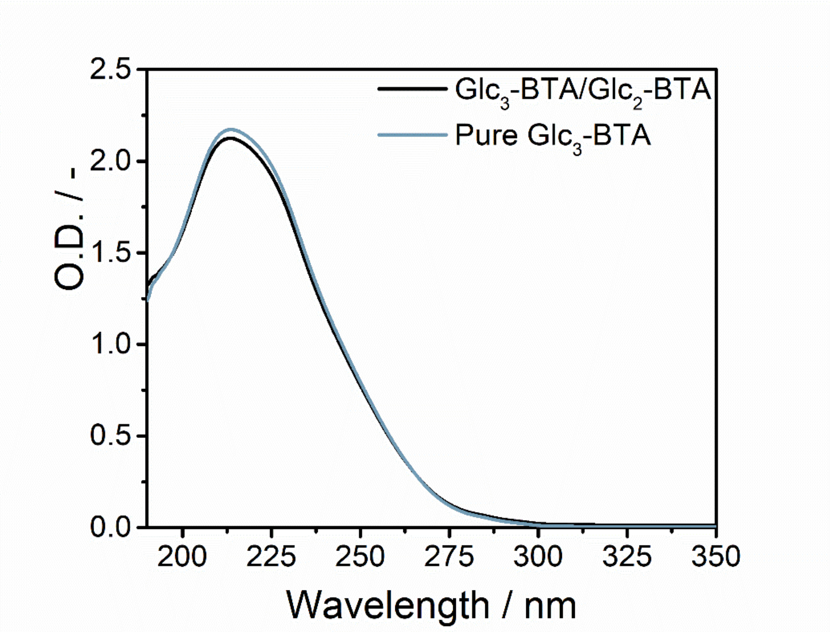

Figure S3: UV spectra of **Glc_3-BTA/Glc_2-BTA** and pure **Glc_3-BTA** after self-assembly in water (c = 50 µM, l = 1 cm, T = 20 °C, bandwidth = 1.0 nm, scan speed = 100 nm/min and data interval = 0.1 nm. All spectra were averaged over three measurements.).

2.1.4 Cryo-TEM Measurements

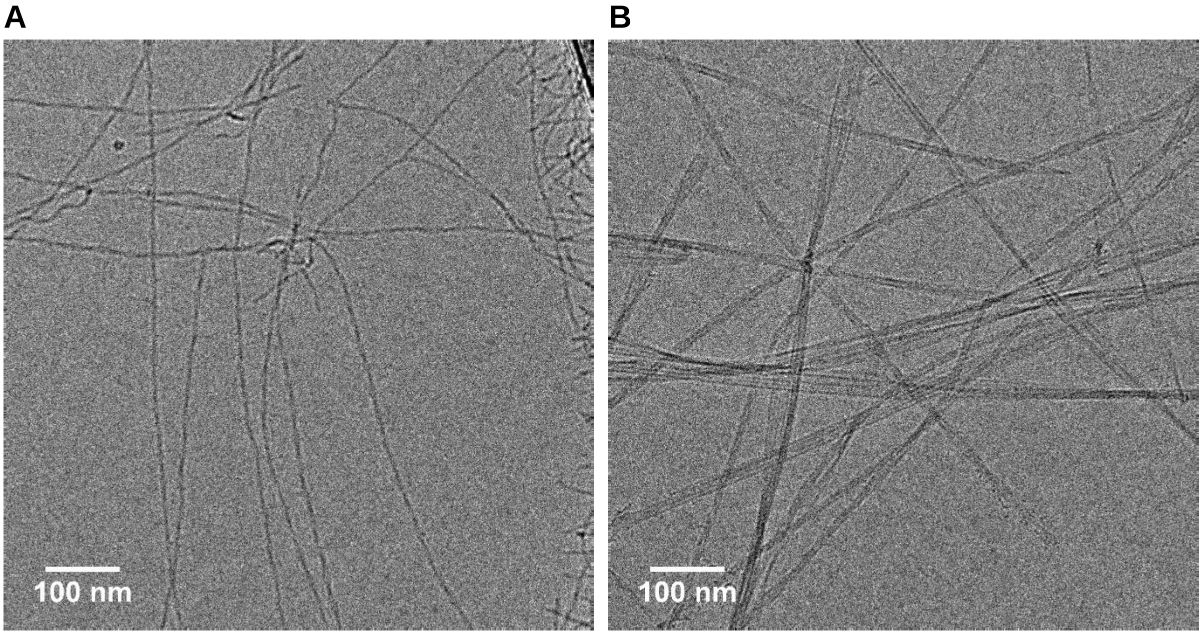

Figure S4: Additional cryo-TEM images of **A) Glc_3-BTA/Glc_2-BTA** and **B)** pure **Glc_3-BTA** (c = 500 µM) showing fiber bundling of **Glc_3-BTA** in absence of **Glc_2-BTA**.

2.1.5 HDX-MS Measurements

The analysis of the HDX-MS measurements is based on a previously published method.[2] All HDX-MS experiments were performed under similar conditions to eliminate influences from the environment, for example temperature. The measurements were performed by diluting a sample of self-assembled supramolecular polymers in H_2O 100-fold into D_2O (including 0.5 mM sodium acetate to facilitate the detection). The distributions of **Glc_3-BTA** with two sodium ions were used for the calculations.

Isotope correction is based on the following set of equations:

$I_{BTA10D\_c} = I_{772.46}$

$I_{BTA11D\_c} = I_{772.96} - 0.87I_{BTA10D\_c}$

$I_{BTA12D\_c} = I_{773.46} - 0.87I_{BTA11D\_c} - 0.42I_{BTA10D\_c}$

$I_{BTA13D\_c} = I_{773.97} - 0.87I_{BTA12D\_c} - 0.42I_{BTA11D\_c} - 0.14I_{BTA10D\_c}$

$I_{BTA14D\_c} = I_{774.47} - 0.87I_{BTA13D\_c} - 0.42I_{BTA12D\_c} - 0.14I_{BTA11D\_c} - 0.04I_{BTA10D\_c}$

$I_{BTA15D\_c} =$

$I_{774.97} - 0.87I_{BTA14D\_c} - 0.42I_{BTA13D\_c} - 0.14I_{BTA12D\_c} - 0.04I_{BTA11D\_c} - 0.01I_{BTA10D\_c}$

with $I_{772.46}$, $I_{772.96}$, $I_{773.46}$, $I_{773.97}$, $I_{774.47}$ and $I_{774.97}$ the intensity at m/z = 772.46, 772.96, 773.46, 773.97, 774.47 and 774.97, respectively.

The correction for the presence of 1 vol% H_2O can be calculated with the following set of equations:

$$I_{BTA10D} = I_{BTA10D\_c} - 3.21\times10^{-7} I_{BTA15D\_c} - 6.71\times10^{-3} I_{BTA12D\_c}$$

$$I_{BTA11D} = I_{BTA11D\_c} - 1.44\times10^{-5} I_{BTA15D\_c} - 1.22\times10^{-1} I_{BTA12D\_c}$$

$$I_{BTA12D} = I_{BTA12D\_c} - 4.74\times10^{-4} I_{BTA15D\_c} + (6.71\times10^{-3} + 1.22\times10^{-1}) I_{BTA12D\_c}$$

$$I_{BTA13D} = I_{BTA13D\_c} - 1.08\times10^{-2} I_{BTA15D\_c}$$

$$I_{BTA14D} = I_{BTA14D\_c} - 1.52\times10^{-1} I_{BTA15D\_c}$$

$$I_{BTA15D} = I_{BTA15D\_c} + (3.21\times10^{-7} + 1.44\times10^{-5} + 4.74\times10^{-4} + 1.08\times10^{-2} + 1.52\times10^{-1}) I_{BTA15D\_c}$$

The percentage of the deuterated analogues can be calculated:

$$\%BTAnD = \frac{I_{BTAnD}}{\sum_{k=1}^{6} I_{BTAkD}} \times 100\%$$

with $\sum_{k=1}^{6} I_{BTAkD}$ the sum of all intensities. The percentage of deuterated analogues was calculated for several time points and plotted as a function of time.

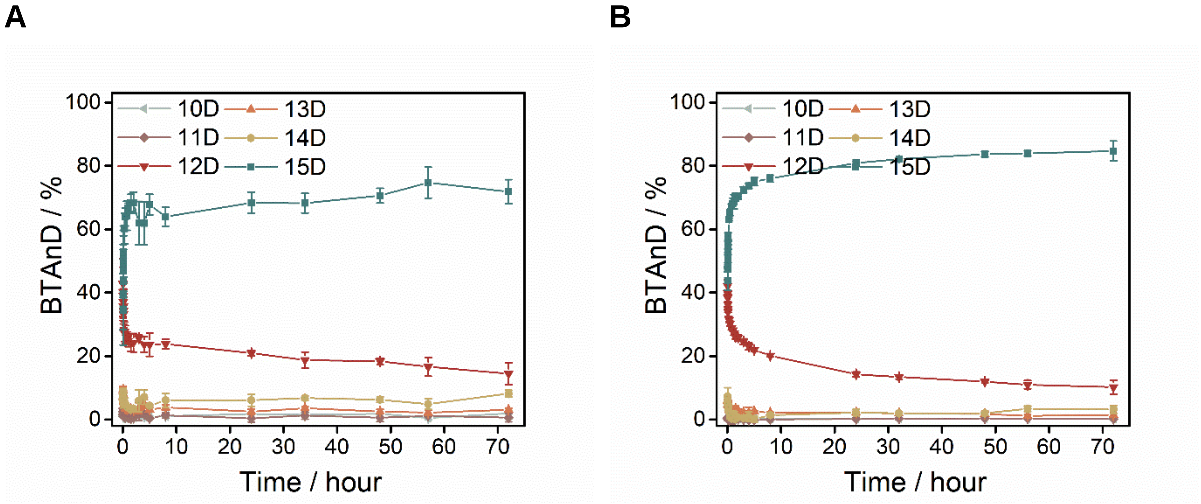

Figure S5: Comparison of the HDX-MS experiments of **A) Glc3-BTA/Glc2-BTA** and **B)** pure **Glc3-BTA**. The percentage of deuterated analogs of **Glc3-BTA** is plotted as a function of time after the 100-fold dilution of an aqueous 500 µM sample into D_2O (T = room temperature). The symbols represent the average and the error bars the standard deviation calculated from 3 independent measurements. Lines are added to guide the eye. The data from A) corresponds to previously published data from reference 3. The higher percentage of fully deuterated BTA (BTA15D) in **B)** indicates that the monomer exchange dynamics of the supramolecular polymers have increased in case of the purer sample.

2.2 Impact of Water on the Self-Assembly of ***n*-TTA** in **(*R*)-CldMeOct**

The compounds and solvents were synthesized as previously published.[4, 5]

2.2.1 Sample Preparation

From each compound a 75 µM stock solution was prepared according to the following procedure: The desired compound was weighed in on an aluminum weighing boat, placed in a 4 mL screw-cap vial and mixed with the according amount of freshly distilled solvent (solvent was kept under inert conditions and distilled into flasks equipped with 3 Å molecular sieves). The samples were flushed with nitrogen and equilibrated while stirring with a magnetic stirring bar in a sand bath at 120 °C for 15 min. After all compound had dissolved, the samples were taken out of the heating bath and cooled to room temperature by leaving them on the bench. The ECD spectra of the freshly prepared samples were directly measured after transferring the samples into a 1 mm screw cap cuvette (the cuvettes were filled and sealed under ambient conditions, creating an "ambient" air buffer above the sample). The change in ECD intensity at 266 nm was monitored over 14 h. The stock solution was kept under inert conditions.

2.2.2 UV-vis Spectroscopic Analysis

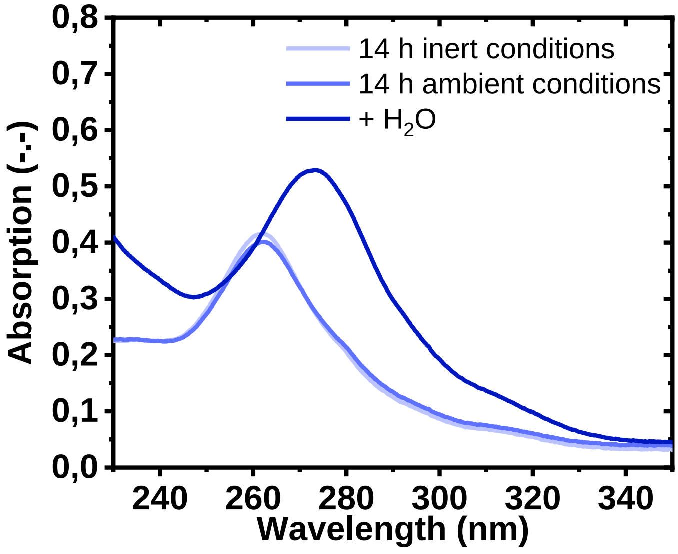

Figure S6: UV spectra of ***n*-TTA** in **(*R*)-CldMeOct** (*c* = 75 µM, *l* = 0.1 cm, *T* = 20 °C, note that these UV trace originate from the ECD spectrometer) when stored for 14 h under inert and ambient conditions and after addition of 3 µL water. The strong red shift in absorption to 276 nm indicates disassembly of the supramolecular polymers.[3]

2.3 UV-vis Spectroscopic Analysis of Different Decalins

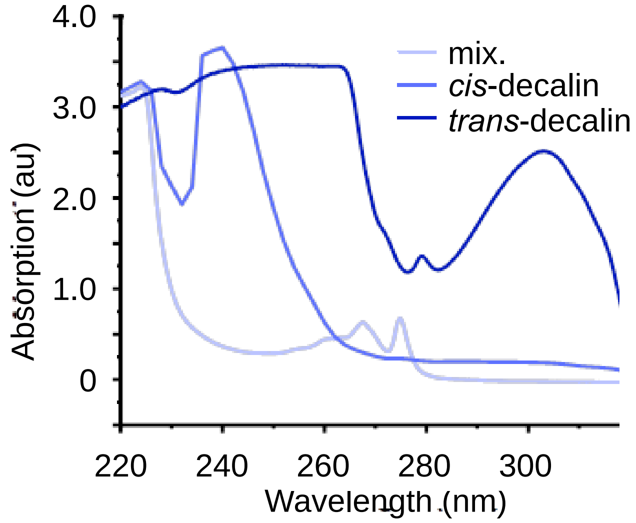

Figure S7: UV spectra of spectroscopic grade *cis*-decalin, *trans*-decalin, and a diastereomeric mixture (l = 1.0 cm, T = 20 °C, note that these UV trace originate from the ECD spectrometer).

3 References

[1] C. M. A. Leenders, G. Jansen, M. M. M. Frissen, R. P. M. Lafleur, I. K. Voets, A. R. A. Palmans, E. W. Meijer, *Chem. Eur. J.* **2016**, *22*, 4608.

[2] X. Lou, S. M. C. Schoenmakers, J. L. J. van Dongen, M. Garcia-Iglesias, N. M. Casellas, M. Fernández-Castaño Romera, R. P. Sijbesma, E. W. Meijer, A. R. A. Palmans, *J. Polym. Sci.* **2021**, *59*, 1151.

[3] S. M. C. Schoenmakers, C. M. A. Leenders, R. O. M. Lafleur, X. Lou, E. W. Meijer, G. M. Pavan, A. R. A. Palmans, *Chem. Commun.* **2018**, *54*, 11128.

[4] M. L. Ślęczkowski, M. F. J. Mabesoone, P. Ślęczkowski, A. R. A. Palmans, E. W. Meijer, *Nat. Chem.* **2021**, *13*, 200.

[5] A. K. Mondal, M. D. Preuss, M. L. Ślęczkowski, T. K. Das, G. Vantomme, E. W. Meijer, R. Naaman *J. Am. Chem. Soc.* **2021**, *143*, 7189.
